# Evaluating the effectiveness of intravenous alteplase in patients with minor non-disabling stroke and severe large vessel stenosis: a retrospective study

**DOI:** 10.3389/fneur.2025.1661549

**Published:** 2025-10-03

**Authors:** Qiong Du, Xiaoxuan Li, Yangli Tang, Xuemei Li, Xijun Li, Haichun Hou, Yang Yan, Xichang Liu, Zhengbiao Huang, Zhenxing Liu, Hailong Xu

**Affiliations:** ^1^Department of Neurology, Yichang Yiling People's Hospital, Yichang, China; ^2^Department of Neurology, Renhe Hospital Affiliated to Three Gorges University, Yichang, China; ^3^Department of Neurology, Sinopharm Gezhouba Central Hospital, Yichang, China

**Keywords:** minor non-disabling, acute ischemic stroke, large vessel stenosis, thrombolysis, alteplase

## Abstract

**Aim:**

The efficacy of intravenous alteplase thrombolysis in patients with minor non-disabling acute ischemic stroke with severe large vessel stenosis or occlusion remains uncertain. This study aimed to assess the clinical effectiveness of intravenous alteplase therapy compared to dual antiplatelet therapy (DAPT) in this specific high-risk population.

**Methods:**

We conducted a retrospective study of patients presenting with minor non-disabling strokes [National Institutes of Health Stroke Scale (NIHSS) score ≤ 5 with all item scores 0 or 1] and severe large vessel stenosis or occlusion across four hospitals in Yichang City, Hubei Province (January 2019–December 2024, *n* = 396). Propensity score matching (1:1 nearest-neighbor with caliper 0.2 SD) was performed to balance baseline characteristics. Multivariable logistic regression models with progressive adjustment for demographic, clinical, laboratory, and imaging variables were used to evaluate the association between the treatment allocation (intravenous alteplase vs. DAPT) and excellent functional outcomes [modified Rankin Scale (mRS) 0–1 at 90 days].

**Results:**

Among 396 included patients (median age 68 years, 67.9% male), 199 received alteplase treatment and 197 received DAPT. The alteplase group demonstrated significantly higher rates of excellent functional outcomes at 90 days compared to the DAPT group (79.9 vs. 56.9%, *p* < 0.001). with adjusted odds ratio of 3.17 (95% CI: 1.77–5.66) in the fully adjusted model. Conditional logistic regression analysis in the propensity score-matched cohort consistently showed superior efficacy of alteplase (OR: 3.24, 95% CI: 1.75–5.98). Safety outcomes did not differ significantly between groups with comparable rates of hemorrhagic transformation (3.0 vs 1.5%, *p* = 0.503), and symptomatic intracranial hemorrhage (1.5 vs. 0%, *p* = 0.248).

**Conclusion:**

In summary, for patients with minor non-disabling stroke and severe large vessel stenosis or occlusion, intravenous alteplase administered within 4.5 h is associated with significantly better functional outcomes compared to DAPT, without increasing the risk of hemorrhagic complications. These findings support the use of thrombolysis in this specific patient population and highlight the need for randomized controlled trials to confirm these results.

## 1 Introduction

Minor non-disabling stroke is characterized by relatively mild clinical symptoms, and typically defined as a National Institutes of Health Stroke Scale (NIHSS) scores ≤ 5, with all scoring items are rated either 0 or 1 (with items 1a to 1c scoring 0) (1, 2). Large vessel stenosis or occlusion significantly is a major cause of ischemic stroke, with approximately 10% of patients who experience minor stroke. Among those who do not receive reperfusion therapy (a treatment to restore blood flow), about 20% will experience neurological deterioration (3, 4). Early neurological decline in minor strokes caused by large vessel occlusion can be attributed to several mechanisms: inadequate collateral blood flow, thrombus propagation, secondary thromboembolism, and the enlargement of the initially ischemic region (5, 6).

Current guidelines recommend intravenous thrombolysis for minor strokes that cause disabilities (7, 8). The 2019 AHA/ASA Guidelines specifically advise against intravenous thrombolysis for non-disabling minor stroke irrespective of time window (7). In contrast, the 2021 European Thrombolysis Guidelines offer a weak recommendation (supported by 6/8 experts) for intravenous thrombolysis in non-disabling minor stroke with confirmed large vessel occlusion (LVO) within 4.5 h of onset (8). This clinical uncertainty is further reflected in recent trials. The 2023 *Antiplatelet* vs. *Rt-PA for Acute Mild Ischemic Stroke* (ARAMIS) trial (1) enrolled 760 individuals with acute mild non-disabling ischemic stroke to examine whether dual antiplatelet therapy (DAPT) was non-inferior to intravenous thrombolysis using alteplase within 4.5 h of onset. The results demonstrated that for patients with acute mild non-disabling ischemic stroke within 4.5 h of onset, DAPT produced 90-day functional outcomes non-inferior to those achieved with intravenous alteplase. The *Potential of Rt-PA for Ischemic Strokes With Mild Symptoms* (PRISMS) trial (9), which was terminated early for futility, found no significant difference in the functional outcomes at the 90-day between the alteplase and aspirin alone, but identified a significantly higher risk of symptomatic intracerebral hemorrhage (sICH) in the alteplase group.

Currently, there are no clear diagnostic and treatment guidelines for acute mild stroke with large vessel occlusion. Studies demonstrates that patients with minor strokes due to stenosis or occlusion of large blood vessels frequently experience early neurological decline and poor recovery. This may occur due to stroke recurrence or the enlargement of the infarct core despite medical medical therapy (10–13). This clinical challenge warrants investigation of reperfusion therapies for such patients. Our study aims to conduct an in-depth analysis of the clinical effects of intravenous thrombolysis in patients with mild non-disabling strokes combined with severe large vessel stenosis or occlusion, in order to provide new insights into treatment strategies in this field.

## 2 Methods

### 2.1 Study population and design

This study was reported following the Strengthening the Reporting of Observational Studies in Epidemiology (STROBE) guidelines. A completed STROBE checklist is provided as Supplementary material.

We retrospectively enrolled individuals with mild non-disabling stroke and severe stenosis or occlusion of large vessels. Patients were recruited from four medical centers, namely Yiling People's Hospital of Yichang, Yichang First People's Hospital, Renhe Hospital Affiliated to Three Gorges University, and Sinopharm Gezhouba Central Hospital (January 2019–December 2024). We consecutively screened and enrolled all eligible patients during the study period. Inclusion criteria were as follows: (1) diagnosed with a mild non-disabling stroke: NIHSS score ≤ 5, and each baseline NIHSS score item scored 0 or 1 (items 1a to 1c scored 0); (2) severe stenosis or occlusion of the intracranial/internal carotid artery, middle cerebral artery (M1/M2), anterior cerebral artery (A1), posterior cerebral artery (P1), vertebral artery, or basilar artery, confirmed by magnetic resonance angiography (MRA), computed tomography angiography (CTA), or digital subtraction angiography (DSA); (3) no subsequent endovascular therapy; (4) treatment initiated within 4.5 h of symptom onset with either: intravenous recombinant tissue plasminogen activator (rt-PA: 0.9 mg/kg, maximum 90 mg; 10% bolus, 90% over 1 h); DAPT consisted of aspirin and clopidogrel (aspirin at a dosage of 100 mg per day, along with an initial clopidogrel dose of either 300 mg, followed by a maintenance dose of 75 mg daily); and (5) modified Rankin Scale (mRS) score ≤ 1 before stroke onset. Patients were excluded if they: (1) brain tumor; (2) severe renal (eGFR <30 ml/min/1.73 m^2^), or hepatic impairment (Child-Pugh C); (3) severe end-stage disease (life expectancy <6 months); (4) atrial fibrillation; (5) lost to follow-up at 90 days. The local human ethics committee (Yiling People's Hospital of Yichang Ethics Committee) granted approval for this study. Additionally, written informed consent was secured from all participants or legal representatives.

### 2.2 Data collection

Patients were categorized into intravenous alteplase or DAPT groups based on initial treatment. We systematically collected various types of information for each group, including demographic data (age, sex, height, and weight), medical history (smoking, drinking, diabetes, hypertension, and coronary heart disease), stroke characteristics (baseline NIHSS score: range 0–42, with higher scores indicating more severe stroke), vascular territory (anterior/posterior circulation), white matter lesion grading (Fazekas grading: 0–3 levels), and laboratory parameters (random blood glucose, white blood cells, platelet count, neutrophils, lymphocytes, total cholesterol, triglycerides, low-density lipoprotein, high-density lipoprotein, alanine aminotransferase, aspartate aminotransferase, total bilirubin, albumin, urea nitrogen, creatinine, homocysteine, D-dimer, and fibrinogen). The primary outcome of the investigation was favorable functional outcome defined as mRS score 0–1 at 90 days. Certified vascular neurologists blinded to treatment allocation assessed 90-day mRS scores using validated telephone interviews. The hemorrhagic transformation was evaluated according to ECASS-III criteria on 24-h follow-up neuroimaging. All variables collected were considered potential predictors or confounders based on previous literature and clinical knowledge. These variables were adjusted for in the multivariable analyses to isolate the independent treatment effect on clinical outcomes.

To address potential sources of bias, we implemented the following strategies: (1) assessment blinding: outcome assessors were blinded to treatment allocation to minimize information bias. (2) Minimizing attrition: we employed rigorous follow-up protocols to reduce selection bias due to loss to follow-up. (3) Controlling for confounding: in the statistical analysis, we constructed multivariable regression models to adjust for a wide range of potential confounding variables (see Statistical analysis section for details).

### 2.3 Imaging assessment

Vascular stenosis severity was classified based on CTA, MRA, or DSA as follows: normal (0% stenosis), mild to moderate (greater than 0% but less than 70%), severe (70% to less than 100%), and complete occlusion (100% stenosis) (14). A brain computed tomography (CT) or magnetic resonance imaging (MRI) scan was conducted again 24 h post-treatment to assess for any occurrence of hemorrhagic transformation. Symptomatic intracranial hemorrhage (sICH) was characterized based on the criteria established by the European Cooperative Acute Stroke Study (ECASS) III. This classification encompasses remote parenchymal hemorrhage type 2, subarachnoid hemorrhage, or intraventricular hemorrhage associated with a neurologic decline of 4 points or greater on the NIHSS from baseline or the lowest NIHSS score between baseline and the deterioration, or resulting in mortality (15). The Fazekas grading scale quantifies white matter hyperintensity severity on T2-weighted or FLAIR MRI sequences. It is divided into four grades: Fazekas grade 0: no white matter lesions; Fazekas grade 1: punctate lesions; Fazekas grade 2: confluent periventricular lesions; Fazekas grade 3: diffuse white matter involvement (16, 17).

### 2.4 Statistical analysis

All analyses were conducted utilizing R software (version 4.3.1, accessible at https://www.r-project.org/). The “MatchIt” package (version 4.5.5) was used for propensity score matching, and the “survival” package (version 3.5.7) was employed for conditional logistic regression on the matched cohort. The dataset was complete for all variables used in the models, with no missing values.

Continuous variables were expressed as mean ± standard deviation (SD) or median with interquartile range (IQR) based on their distribution as assessed by the Shapiro–Wilk test. Categorical variables were presented as frequencies with percentages. Group comparisons in the original (unmatched) cohort used the Student *t*-test for normally distributed continuous variables, the Mann–Whitney *U* test for non-normally distributed continuous variables, and the χ^2^ test or Fisher's exact test (for expected cell counts <5) for categorical variables.

PSM was performed to minimize selection bias. The propensity score was estimated using a logistic regression model that included the following baseline covariates: age, sex, baseline NIHSS score, hypertension and diabetes mellitus, smoking status, and drinking habit. A 1:1 nearest-neighbor matching algorithm, with a caliper width of 0.2 standard deviations of the logit propensity score, was used to construct the matched cohort. Covariate balance was assessed using standardized mean differences (SMD), where an SMD <0.1 indicated good balance. Following matching, all covariates achieved satisfactory balance (all SMDs <0.1; detailed in Supplementary Table S1). For group comparisons within the matched cohort, we employed paired statistical tests—including the paired *t*-test, Wilcoxon signed-rank test, and McNemar's test—as appropriate for the data type.

Logistic regression analyses, both univariate and multivariable, were conducted to evaluate the association between treatment allocation and clinical outcomes. Variables were specified as follows: the baseline NIHSS score and all laboratory parameters were treated as continuous variables; the Fazekas score was incorporated as an ordinal variable (range 0–3); and all other categorical variables were included in their original form.

We constructed four sequential multivariable logistic regression models to evaluate the independent association between the treatment and outcomes, with progressive adjustment for clusters of potential confounders selected *a priori* based on established prognostic relevance and clinical grounds (18).

The primary analysis was conducted on the overall cohort using sequential models:

Model 1 (Overall Cohort): unadjusted;
Model 2 (Overall Cohort): adjusted for core demographic factors: age and sex;
Model 3 (Overall Cohort): additionally adjusted for BMI, smoking status, alcohol consumption, hypertension, diabetes mellitus, coronary heart disease (CHD), responsible vessel location, and Fazekas score;
Model 4 (Overall Cohort): further adjusted for baseline NIHSS score, glucose, total cholesterol, triglycerides, high-density lipoprotein, low-density lipoprotein, alanine aminotransferase, blood urea nitrogen, creatinine, homocysteine, D-dimer, fibrinogen, neutrophil-to-lymphocyte ratio (NLR), and hemorrhagic transformation. This model aimed to isolate the treatment effect by controlling for disease severity, metabolic profile, organ function, thrombotic/inflammatory state, and a key safety outcome.

A supplementary analysis was conducted on the matched cohort using conditional logistic regression:

Model 5 (Matched Cohort): conditional model containing only the treatment variable;
Model 6 (Matched Cohort): additionally adjusted for diabetes mellitus, glucose, NLR, and fibrinogen-variables selected for their established biological relevance to the outcome.

Odds ratios (ORs) with 95% confidence intervals (CIs) were computed. All statistical tests were two-sided, with a significance threshold set at *p* < 0.05.

## 3 Results

**Figure 1:**
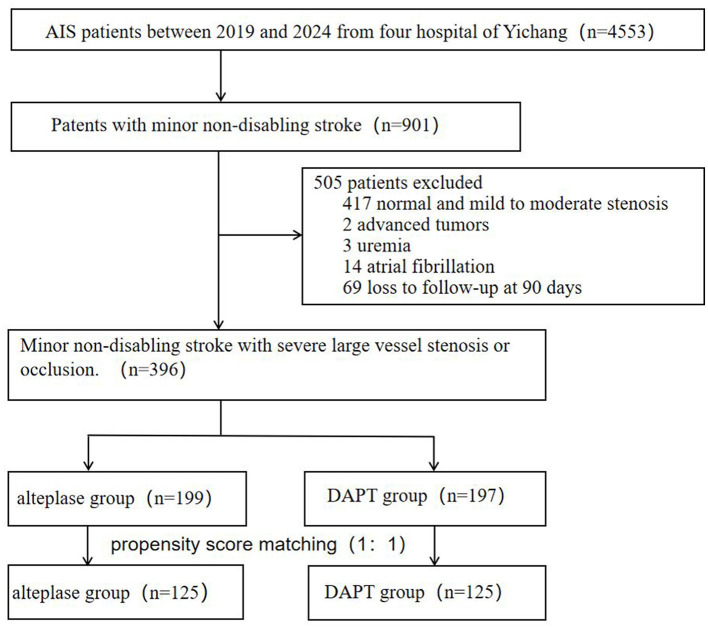
Study flowchart showing the recruitment and inclusion of eligible participants.

From 4,553 consecutive stroke patients screened (January 2019–December 2024), 901 presented with mild non-disabling strokes. A total of 505 patients were excluded from the study (as shown in Figure 1), 417 normal or mild to moderate stenosis, two for advanced tumors, three for uremia, 14 atrial fibrillation and 69 due to lost to 90-day follow-up. The final cohort comprised 396 patients: 199 received intravenous alteplase thrombolysis and 197 treated with DAPT.

Baseline characteristics are summarized in Table 1. Compared to the DAPT group, the alteplase group were younger (67 vs. 68 years, *p* = 0.009), had a lower proportion of males (62.8 vs. 73.1%, *p* = 0.037), and demonstrated lower rates of smoking (36.7 vs. 49.7%, *p* = 0.012), and alcohol consumption (27.6 vs. 44.7%, *p* = 0.001). The alteplase group also showed lower baseline NIHSS score [median 2 (IQR 1–2.5) vs. 2 (1–3), *p* = 0.014], reduced neutrophil count/lymphocyte count ratio (NLR, 2.4 vs. 2.9, *p* = 0.003), lower homocysteine levels (13.1 vs. 15.8 μmol/L, *p* < 0.001), lower fibrinogen levels (3.0 vs. 3.5 g/L, *p* < 0.001), and higher D-dimer levels (0.6 vs. 0.3 μg/ml, *p* < 0.001). Significant differences were observed in the proportion of anterior and posterior circulation (*p* = 0.009) as well as Fazekas scores for white matter lesions (*p* < 0.001). No significant difference was found in the rate of hemorrhagic transformation (alteplase vs. DAPT: 6 vs. 3 events, *p* = 0.503). Symptomatic intracranial hemorrhage occurred in three patients (1.5%) in the alteplase group vs. 0 patient (0%) in the DAPT group (*p* = 0.248). No significant differences were found in other variables. Data were complete for all variables presented in this table; there were no missing values. Following propensity score matching, baseline characteristics were well-balanced between the two treatment groups for all variables except D-dimer and fibrinogen levels, which remained significantly different (all standardized mean differences <0.1 except for these two biomarkers).

**Table 1:** Baseline characteristics of patients based on the modalities of medications.

	**Unmatched**			**Matched**		
**Variables**	**Intravenous t-PA (*****n*** = **199)**	**DAPT (*****n*** = **197)**	***p*** **Value**	**Intravenous t-PA (*****n*** = **125)**	**DAPT (*****n*** = **125)**	***p*** **Value**
Age, years	67.0 (59.0–73.0)	68.0 (63.0–76.0)	0.009	67.6 (10.5)	67.0 (9.8)	0.624
Female	74 (37.2%)	53 (26.9%)	0.037	86 (68.8%)	83 (66.4%)	0.787
Smoking	73 (36.7%)	98 (49.7%)	0.012	57 (45.6%)	53 (42.4%)	0.702
Drinking	55 (27.6%)	88 (44.7%)	0.001	45 (36.0%)	47 (37.6%)	0.896
Diabetes mellitus	52 (26.1%)	64 (32.5%)	0.201	39 (31.2%)	39 (31.2%)	1.000
Hypertension	154 (77.4%)	155 (78.7%)	0.850	101 (80.8%)	100 (80.0%)	1.000
Prior CHD/MI	49 (24.6%)	38 (19.3%)	0.246	26 (20.8%)	27 (21.6%)	1.000
Baseline NIHSS	2.0 (1.0–2.5)	2.0 (1.0–3.0)	0.014	2.0 (1.0–2.0)	2.0 (1.0–3.0)	0.378
**Location of responsible vessel**						
Anterior circulation	88 (44.2%)	98 (49.7%)	0.009	54 (43.2%)	62 (49.6%)	0.235
Posterior circulation	95 (47.7%)	96 (48.7%)		63 (50.4%)	60 (48.0%)	
Anterior and posterior circulation	16 (8.0%)	3 (1.5%)		8 (6.4%)	3 (2.4%)	
**Fazekas score**						
0	64 (32.2%)	27 (13.7%)	<0.001	26 (20.8%)	25 (20.0%)	0.901
1	89 (44.7%)	91 (46.2%)		61 (48.8%)	64 (51.2%)	
2	27 (13.6%)	59 (29.9%)		26 (20.8%)	22 (17.6%)	
3	19 (9.5%)	20 (10.2%)		12 (9.6%)	14 (11.2%)	
BMI	23.9 (21.3–25.7)	23.5 (21.4–25.4)	0.981	23.8 (21.0–25.4)	24.0 (21.8–26.0)	0.207
**Laboratory findings**						
Random blood glucose, mmol/L	5.8 (5.0–7.2)	5.5 (5.0–7.1)	0.412	5.9 (5.0–7.5)	5.5 (5.1–7.1)	0.278
Leukocyte count × 10^9^/L	6.7 (5.4–8.1)	6.6 (5.3–8.4)	0.868	6.4 (5.3–7.9)	7.0 (5.5–8.2)	0.285
Platelet count × 10^9^/L	195.0 (166.5–231.5)	187.0 (150.0–231.0)	0.130	186.0 (165.0–219.0)	191.0 (155.0–231.0)	0.619
Neutral lymph ratio	2.4 (1.7–3.9)	2.9 (2.0–4.7)	0.003	2.5 (1.8–4.0)	2.9 (1.9–4.5)	0.117
Total cholesterol, mmol/L	4.3 (3.7–4.9)	4.3 (3.7–5.0)	0.572	4.2 (3.6–4.7)	4.3 (3.8–5.1)	0.243
Triacylglycerol, mmol/L	1.4 (1.0–1.9)	1.2 (0.8–1.8)	0.090	1.4 (1.0–1.9)	1.2 (0.9–1.8)	0.426
HDL cholesterol, mmol/L	1.1 (0.9–1.3)	1.1 (0.9–1.3)	0.723	1.1 (0.9–1.3)	1.1 (0.9; 1.3)	0.597
LDL cholesterol, mmol/L	2.5 (2.0–3.1)	2.6 (2.1–3.1)	0.432	2.4 (2.0–3.0)	2.6 (2.1–3.2)	0.093
ALT, U/L	16.3 (11.8–22.0)	16.4 (12.2–22.2)	0.423	15.4 (11.7–21.3)	16.4 (12.1–23.0)	0.216
AST, U/L	18.2 (15.9–22.3)	18.9 (15.6–24.1)	0.606	19.0 (16.0–22.0)	18.5 (15.0–23.5)	0.722
TBIL, μmol/L	11.7 (8.5–15.1)	11.7 (9.3–15.8)	0.207	12.3 (9.0–15.2)	11.3 (9.1–14.9)	0.230
ALB, g/L	40.3 (37.8–42.5)	39.5 (37.5–41)	0.092	40.1 (3.6)	39.6 (3.7)	0.264
BUN, mmol/L	5.6 (4.4–6.6)	5.7 (4.7–7.2)	0.082	5.7 (4.4–6.7)	5.5 (4.6–7.2)	0.887
Serum creatinine, μmol/L	72.1 (61.0–86.9)	74.0 (64.0–90.0)	0.254	76.7 (62.1–88.0)	76.0 (61.8–89.0)	0.746
HCY, μmol/L	13.1 (10.5–17.1)	15.8 (12.2–23.4)	<0.001	14.0 (10.5–18.4)	14.9 (11.6–21.4)	0.037
D-demer, μg/ml	0.6 (0.3–1.1)	0.3 (0.1–0.5)	<0.001	0.7 (0.3–1.1)	0.3 (0.1–0.4)	<0.001
Fibrinogen, g/L	3.0 (2.5–3.5)	3.5 (3.0–4.1)	<0.001	2.9 (2.5–3.5)	3.5 (3.0–4.1)	<0.001
Hemorrhagic transformation at 24 h,%	6 (3.0%)	3 (1.5%)	0.503	3 (2.4%)	2 (1.6%)	1.000
Symptomatic intracranial hemorrhage at 24 h, %	3 (1.5%)	0 (0.0%)	0.248	1 (0.8%)	0 (0.0%)	1.000

NIHSS, National Institutes of Health Stroke Scale score; ALT, glutamic-pyruvic transaminase; AST, glutamic oxalacetic Transaminase; TBIL, total bilirubin; ALB, albumin; BUN, blood urea nitrogen; HCY, homocysteine; BMI, Weight (kg)/Height (m)^2^; NLR, Neutrophil count/Lymphocyte count, Symptomatic intracranial hemorrhage, bleeding conversion associated with an increase of ≥4 points in the NIHSS score compared to the baseline assessment.

Outcomes are shown in Table 2. In the overall cohort, patients achieving favorable outcomes (mRS 0–1) were significantly younger than those with poor outcomes (67 vs. 71 years, *p* < 0.001) and demonstrated lower NLR (2.5 vs. 3.0, *p* = 0.040), reduced homocysteine (13.8 vs. 16.4 μmol/L, *p* = 0.009) and lower fibrinogen levels (3.3 vs. 3.5 g/L, *p* = 0.013). This pattern was confirmed and extended in the propensity score-matched analysis, which additionally identified a lower prevalence of diabetes mellitus, lower initial glucose levels, and a reduced NLR in the favorable outcomes group. In the DAPT group, 43.1% (85/197) of patients had poor functional outcomes (mRS 2–6), compared to 56.9% (112/197) achieving favorable outcomes (mRS 0–1). Conversely, in the alteplase group, only 20.1% (40/199) had poor outcomes (mRS 2–6), while 79.9% (159/199) attained favorable outcomes (mRS 0–1).

**Table 2:** Univariate comparison of characteristics in patients with minor non-disabling acute ischemic stroke with large vessel severe stenosis or occlusion.

**Variables**	**Unmatched**			**Matched**		
	**mRS 0–1 (*****n*** = **271)**	**mRS 2–6 (*****n*** = **125)**	***p*** **Value**	**mRS 0–1 (*****n*** = **172)**	**mRS 2–6 (*****n*** = **78)**	***p*** **Value**
Age, years	67 (59–73)	71 (64–78)	<0.001	66.7 ± 10.1	68.7 ± 10.3	0.155
Female	88 (32.5%)	39 (31.2%)	0.892	117 (68.0%)	52 (66.7%)	0.947
Smoking	112 (41.3%)	59 (47.2%)	0.324	74 (43.0%)	36 (46.2%)	0.746
Drinking	97 (35.8%)	46 (36.8%)	0.935	64 (37.2%)	28 (35.9%)	0.954
Diabetes mellitus	71 (26.2%)	45 (36.0%)	0.061	46 (26.7%)	32 (41.0%)	0.035
Hypertension	207 (76.4%)	102 (81.6%)	0.301	138 (80.2%)	63 (80.8%)	1.000
Prior CHD/MI	52 (19.2%)	35 (28.0%)	0.066	33 (19.2%)	20 (25.6%)	0.322
Baseline NIHSS	2.0 (1.0–2.0)	2.0 (1.0–3.0)	0.067	2.0 (1.0–3.0)	2.0 (1.0–3.0)	0.088
**Location of responsible vessel**						
Anterior circulation	122 (45.0%)	64 (51.2%)	0.222	79 (45.9%)	37 (47.4%)	0.727
Posterior circulation	133 (49.1%)	58 (46.4%)		84 (48.8%)	39 (50.0%)	
Anterior and posterior circulation	16 (5.9%)	3 (2.4%)		9 (5.2%)	2 (2.6%)	
**Fazekas_score:**						
0	70 (25.8%)	21 (16.8%)	0.062	36 (20.9%)	15 (19.2%)	0.362
1	124 (45.8%)	56 (44.8%)		87 (50.6%)	38 (48.7%)	
2	56 (20.7%)	30 (24.0%)		35 (20.3%)	13 (16.7%)	
3	21 (7.7%)	18 (14.4%)		14 (8.1%)	12 (15.4%)	
BMI	23.9 (21.5–25.7)	23.4 (21.2–25.1)	0.217	23.9 (21.4–25.7)	23.7 (21.4–26.0)	0.876
**Laboratory findings**						
Random blood glucose, mmol/L	5.6 (5.0–6.8)	5.9 (5.0–8.3)	0.083	5.5 (4.9–6.8)	6.0 (5.4–8.5)	0.011
Leukocyte count × 10^9^/L	6.7 (5.3–8.5)	6.6 (5.3–7.8)	0.477	6.7 (5.4–8.1)	6.7 (5.4–8.1)	0.754
Platelet count × 10^9^/L	201.9 ± 62.9	196.5 ± 61.3	0.425	189.5 (159.8–224.0)	182.5 (154.2–231.0)	0.759
Neutral lymph ratio	3.6 ± 3.4	4.5 ± 6.3	0.130	2.5 (1.8–4.0)	3.1 (1.9–5.3)	0.021
Total cholesterol, mmol/L	4.4 ± 1.0	4.5 (1.1)	0.309	4.2 (3.7–4.9)	4.3 (3.7–5.0)	0.918
Triacylglycerol, mmol/L	1.3 (0.9–1.8)	1.2 (0.8–2.0)	0.950	1.3 (1.0–1.9)	1.2 (0.8–1.8)	0.151
HDL cholesterol, mmol/L	1.1 ± 0.3	1.2 ± 0.4	0.490	1.1 (0.9–1.3)	1.2 (1.0–1.3)	0.390
LDL cholesterol, mmol/L	2.6 ± 0.8	2.6 ± 0.8	0.650	2.5 (2.1–3.1)	2.6 (2.1–3.0)	0.850
ALT, U/L	16.7 (12.0–22.6)	15.5 (11.6–20.7)	0.187	16.3 (12.0–22.8)	15.2 (11.7–20.8)	0.500
AST, U/L	19.0 (16.0–22.7)	18.2 (15.1–24.4)	0.922	19.0 (16.0–22.0)	18.0 (15.0–24.0)	0.337
TBIL, μmol/L	11.8 (8.8–15.2)	11.6 (9.1–15.3)	0.836	11.8 (8.7–15.1)	11.6 (9.3–14.9)	0.666
ALB, g/L	40.2 ± 3.5	39.6 ± 4.1	0.113	40.0 ± 3.5	39.6 ± 4.0	0.476
BUN, mmol/L	5.6 (4.5–6.8)	5.6 (4.6–7.2)	0.602	5.6 (4.5–6.9)	5.5 (4.5–6.7)	0.591
Serum creatinine, μmol/L	73.0 (61.0–87.9)	73.7 (63.4–91.0)	0.555	77.7 (61.0–89.6)	72.0 (64.0–86.3)	0.742
HCY, μmol/L	13.8 (11.0–18.7)	16.4 (12.2–21.0)	0.009	14.1 (11.1–18.7)	16.1 (10.9–21.0)	0.229
D-demer, μg/ml	0.4 (0.2–0.8)	0.4 (0.2–0.9)	0.289	0.4 (0.2–0.9)	0.3 (0.2–0.8)	0.982
Fibrinogen, g/L	3.3 ± 0.9	3.5 ± 0.9	0.013	3.1 (2.7–3.7)	3.5 (2.9–4.1)	0.019
Hemorrhagic transformation at 24 h,%	8 (3.0%)	1 (0.8%)	0.283	5 (2.9%)	0 (0.0%)	0.329
Symptomatic intracranial hemorrhage at 24 h, %	2 (0.7%)	1 (0.8%)	1.000	1 (0.6%)	0 (0.0%)	1.000

mRS, modified Rankin Scale; NIHSS, National Institutes of Health Stroke Scale score; ALT, glutamic-pyruvic transaminase; AST, glutamic oxalacetic transaminase; TBIL, total bilirubin; ALB, albumin; BUN, blood urea nitrogen; HCY, homocysteine; BMI, Weight (kg)/Height (m)^2^.

Symptomatic intracranial hemorrhage, bleeding conversion associated with an increase of ≥4 points in the NIHSS score compared to the baseline assessment.

Logistic regression results are presented in Table 3. Univariate logistic regression revealed a strong association between alteplase and reduced risk of poor outcomes (the unadjusted OR = 3.02; 95% CI: 1.93–4.72; *p* < 0.001). This association remained robust across progressively adjusted multivariable models:

**Table 3:** Comparison of main outcomes in patients with two different modalities of medicat in four models.

**Unmatched**						
**Group**	**mRS 2–6**	**mRS 0–1**	**Unadjusted, OR (95%CI)** ***p*** **Value**	**Multivariable adjusted, OR (95%CI)** ***p*** **Value**		
			**Model1** ^*^	**Model2** ^†^	**Model3** ^#^	**Model4** ^&^
DAPT-Treated (%)	85 (43.1%)	112 (56.9%)	Reference	Reference	Reference	Reference
Alteplase-Treated (%)	40 (20.1%)	159 (79.9%)	3.02 (1.93–4.72) *p* < 0.001	2.74 (1.73–4.34) *p* < 0.001	2.87 (1.73–4.76) *p* < 0.001	3.17 (1.77–5.66) *p* < 0.001
**Matched**						
**Group**	**mRS 2–6**	**mRS 0–1**	**Unadjusted, OR (95%CI)** ***p*** **Value**	**Adjusted, OR (95%CI)** ***p*** **Value**		
			**Model5** ^‡^	**Model6** ^‡‡^		
DAPT-Treated (%)	54 (43.2%)	71 (56.8%)	Reference	Reference		
Alteplase-Treated (%)	24 (19.2%)	101 (80.8%)	3.2 (1.81–5.65) *p* < 0.001	3.24 (1.75–5.98) *p* < 0.001		

^*^Model 1: non-adjusted.

^†^Model 2: adjusted for age and sex.

^#^Model 3: adjusted for age, sex, BMI, smoking, drinking, hypertension, diabetes mellitus, CHD, location of responsible vessel, fazekas_score.

^&^Model 4: adjusted for age, sex, smoking, drinking, hypertension, diabetes mellitus, CHD, location of responsible vessel, fazekas_score, baseline NIHSS, glucose, TC, TG, HDL, LDL, ALT, BUN, Cr, HCY, DD, Fib, NLR, hemorrhagic transformation.

^‡^Model5: unadjusted conditional model.

^‡‡^Model6: additionally adjusted for diabetes mellitus, glucose, NLR, fibrinogen.

## 4 Discussion

Our study demonstrates that intravenous alteplase administration is associated with superior functional outcomes compared to DAPT in patients with mild, non-disabling strokes and large vessel stenosis or occlusion. Importantly, this therapeutic benefit was achieved without a statistically significant increase in the risk of hemorrhagic transformation, as evidenced by comparable rates between the treatment groups.

Current evidence from randomized trials (PRISMS [terminated for futility] (9) and ARAMIS [demonstrating non-inferiority] (1)) and retrospective analyses (19) generally indicates that intravenous thrombolysis provides no significant efficacy advantage over antiplatelet therapy in unselected patients with mild non-disabling stroke deficits without large vessel occlusion. A recent meta-analysis of 3,975 patients reinforced this conclusion, demonstrating comparable 90-day functional outcomes (mRS 0–1: OR 1.08, 95% CI 0.73–1.58) alongside a fivefold increased risk of sICH with thrombolysis (OR 0.20, 95% CI 0.06–0.69) (20). These findings support current guideline recommendations against routine thrombolysis in this broader population.

Minor strokes without large vessel occlusion generally have a favorable clinical prognosis (21). However, an important high-risk subgroup—patients with underlying LVO, often experience early neurological deterioration and worse clinical outcomes (22, 23). Among patients with acute LVO presenting with mild symptoms, approximately 34% fail to achieve functional independence at 90 days without timely revascularization (24). Symptomatic stenosis ≥50% or occlusion in intracranial or extracranial vessels has been identified as a key predictor of early neurological worsening (25), and once progression occurs, secondary reperfusion therapies provide limited benefit (26, 27). This highlights the critical therapeutic urgency in this specific population, for which our study suggests alteplase may offer significant benefit.

Our findings align with emerging evidence suggesting that patients with mild non-disabling stroke and severe large vessel stenosis or occlusion may represent a distinct subgroup that benefits from thrombolysis. Previous retrospective and prospective studies have indicated improved outcomes with intravenous thrombolysis in such patients (19, 28). Collectively, these data strongly support targeted thrombolysis in this high-risk subgroup. We recommend consideration of active intervention for mild stroke patients with severe large vessel stenosis or occlusion, particularly when revascularization is achievable within therapeutic windows.

The recent TEMPO-2 trial compared tenecteplase vs. antiplatelet therapy in patients with mild stroke and confirmed intracranial LVO (29). While its subgroup analysis showed neutral efficacy across all stroke severity subgroups. it is noteworthy that tenecteplase possesses a distinct pharmacologic profile (longer half-life, higher fibrin specificity) that may explain differential treatment effects compared to alteplase. Thus, the critical question of alteplase efficacy in this specific population remains unresolved, underscoring the need for randomized controlled trials specifically evaluating alteplase in this high-risk cohort.

The safety profile of thrombolysis in mild stroke populations deserves particular attention. Previous reports indicate that the risk of sICH following intravenous thrombolysis in patients with mild strokes ranges from 0 to 5% (11, 30, 31). In our study, alteplase-associated sICH occurred in 1.5% of patients, within this expected range and statistically comparable to DAPT (1.5 vs. 0.5%, *p* = 0.248). This finding reinforces that protocol-guided alteplase administration in this selected population does not significantly increase hemorrhagic risk.

The role of endovascular thrombectomy (EVT) in patients with mild non-disabling strokes and significant stenosis or occlusion of major vessels remains uncertain. The 2019 guidelines from the European Stroke Organization and the European Society of Minimally Invasive Neurological Therapy (32) recommend mechanical thrombectomy only if they progress to a significant deficit or worsen despite intravenous thrombolysis. Notably, no expert consensus was reached regarding EVT for persistently non-disabling stroke presentations. A 2023 retrospective propensity score-matched analysis demonstrated lower rates of 90-day functional independence and higher hemorrhagic complications with EVT compared to intravenous thrombolysis in this population (33). Consequently, current evidence remains insufficient to support routine endovascular thrombectomy in patients with persistently mild non-disabling strokes. Ongoing trials such as ENDOLOW (NCT04167527) and MOSTE (NCT03796468) (34) will provide further evidence regarding EVT in mild strokes. The potential risks associated with EVT may contribute to patient and family hesitancy in clinical settings, highlighting the need for safer alternatives like alteplase. Our data support intravenous thrombolysis as a promising therapeutic approach, potentially mediated through multiple mechanisms: rapid thrombus dissolution with subsequent reperfusion, ischemic penumbra salvage, enhancement of microcirculation, and mitigation of reperfusion injury.

Several limitations of our study warrant consideration. First, the retrospective design may introduce selection bias, underscoring the need for validation through more prospective studies and randomized controlled trials. The direction of this potential bias remains unpredictable; it could either attenuate or exaggerate the observed treatment effect. Second, the limited sample size and recruitment of most patients from a single province (Hubei) constrain the generalizability of our findings. While the magnitude of this potential bias is difficult to quantify precisely, the relative homogeneity of the cohort suggests that that our effect estimates might be less applicable to populations with different demographic or healthcare characteristics. Therefore, larger, multicenter studies are warranted to confirm and extend these results. Third, medication non-adherence, observed as discontinuation of antiplatelet therapy during follow-up in some patients, may have confounded the outcomes. This would likely lead to an underestimation of the true effectiveness of the treatment regimen, as the observed outcomes would be diluted by non-adherent participants. Future studies should implement robust measures to monitor and enhance medication adherence. Therefore, the external validity of our findings may be primarily limited to similar hospital settings within comparable demographic and healthcare contexts, pending further validation.

## 5 Conclusions

In conclusion, intravenous alteplase treatment was associated with significantly superior functional outcomes compared to DAPT in patients with minor non-disabling strokes accompanied by severe stenosis or occlusion of large cerebral vessels. Importantly, no statistically significant increase in the risk of hemorrhagic complications was observed between the treatment groups. These compelling findings necessitate further validation through large-scale, multicenter randomized controlled trials with with larger sample sizes, longer follow-up periods, and standardized outcome measures to definitively establish the comparative clinical benefit and safety profile of thrombolysis in this specific high-risk patient population.

## Data Availability

The original contributions presented in the study are included in the article/Supplementary material, further inquiries can be directed to the corresponding authors.
