# Supplementary material for: Evaluating the effectiveness of intravenous alteplase in patients with minor non-disabling stroke and severe large vessel stenosis: a retrospective study
Source: Front Neurol. 2025 Oct 3;16:1661549. doi: 10.3389/fneur.2025.1661549 (PMC12531376; doi:10.3389/fneur.2025.1661549)
Supplement: Supplementary file 1 [file Table_1.docx]

Supplementary Table S1. Comparison of Standardized Mean Differences (SMD) for Baseline Characteristics Before and After Propensity Score Matching

|  | **Before PSM** | | | | **After PSM** | | | |
| --- | --- | --- | --- | --- | --- | --- | --- | --- |
| Variables | 0  N = 1971 | 1  N = 1991 | Difference2 | 95% CI2 | 0  N = 1251 | 1  N = 1251 | Difference2 | 95% CI2 |
| Age,years | 68.0(63.0,76.0) | 67.0(59.0,73.0) | 0.31 | 0.11, 0.51 | 67.0(62.0,74.0) | 69.0(60.0,75.0) | -0.06 | -0.31, 0.19 |
| female | 144 (73.1%) | 125 (62.8%) | 0.22 | 0.02, 0.42 | 83 (66.4%) | 86 (68.8%) | 0.05 | -0.20, 0.30 |
| smoking | 98 (49.7%) | 73 (36.7%) | 0.27 | 0.07, 0.46 | 53 (42.4%) | 57 (45.6%) | 0.06 | -0.18, 0.31 |
| drinking | 88 (44.7%) | 55 (27.6%) | 0.36 | 0.16, 0.56 | 47 (37.6%) | 45 (36.0%) | 0.03 | -0.21, 0.28 |
| Diabetes | 64 (32.5%) | 52 (26.1%) | 0.14 | -0.06, 0.34 | 39 (31.2%) | 39 (31.2%) | 0.00 | -0.25, 0.25 |
| Hypertension | 155 (78.7%) | 154 (77.4%) | 0.03 | -0.17, 0.23 | 100 (80.0%) | 101 (80.8%) | 0.02 | -0.23, 0.27 |
| CHD | 38 (19.3%) | 49 (24.6%) | 0.13 | -0.07, 0.33 | 27 (21.6%) | 26 (20.8%) | 0.02 | -0.23, 0.27 |
| NIHSS_0 |  |  | -0.25 | -0.44, -0.05 |  |  | -0.11 | -0.36, 0.14 |
| 0 | 44 (22.3%) | 12 (6.0%) |  |  | 27 (21.6%) | 8 (6.4%) |  |  |
| 1 | 52 (26.4%) | 56 (28.1%) |  |  | 30 (24.0%) | 38 (30.4%) |  |  |
| 2 | 45 (22.8%) | 81 (40.7%) |  |  | 27 (21.6%) | 50 (40.0%) |  |  |
| 3 | 41 (20.8%) | 34 (17.1%) |  |  | 30 (24.0%) | 19 (15.2%) |  |  |
| 4 | 12 (6.1%) | 11 (5.5%) |  |  | 8 (6.4%) | 8 (6.4%) |  |  |
| 5 | 3 (1.5%) | 5 (2.5%) |  |  | 3 (2.4%) | 2 (1.6%) |  |  |
| Location of responsible vessel |  |  | 0.31 | 0.11, 0.51 |  |  | 0.22 | -0.03, 0.47 |
| 1 | 98 (49.7%) | 88 (44.2%) |  |  | 62 (49.6%) | 54 (43.2%) |  |  |
| 2 | 96 (48.7%) | 95 (47.7%) |  |  | 60 (48.0%) | 63 (50.4%) |  |  |
| 3 | 3 (1.5%) | 16 (8.0%) |  |  | 3 (2.4%) | 8 (6.4%) |  |  |
| fazekas_score |  |  | 0.54 | 0.34, 0.74 |  |  | 0.10 | -0.15, 0.34 |
| 0 | 27 (13.7%) | 64 (32.2%) |  |  | 25 (20.0%) | 26 (20.8%) |  |  |
| 1 | 91 (46.2%) | 89 (44.7%) |  |  | 64 (51.2%) | 61 (48.8%) |  |  |
| 2 | 59 (29.9%) | 27 (13.6%) |  |  | 22 (17.6%) | 26 (20.8%) |  |  |
| 3 | 20 (10.2%) | 19 (9.5%) |  |  | 14 (11.2%) | 12 (9.6%) |  |  |
| BMI | 23.5(21.4,25.4) | 23.9(21.3,25.7) | 0.01 | -0.18, 0.21 | 24.0(21.8,26.0) | 23.8(21.0,25.4) | 0.18 | -0.06, 0.43 |
| Laboratory findings |  |  |  |  |  |  |  |  |
| glucose | 5.5(5.0,7.1) | 5.8(5.0,7.2) | 0.12 | -0.08, 0.32 | 5.5(5.1,7.1) | 5.9(5.0,7.5) | 0.13 | -0.12, 0.37 |
| WBC | 6.6(5.3,8.4) | 6.7(5.4,8.2) | 0.00 | -0.19, 0.20 | 7.0(5.5,8.2) | 6.4(5.3,7.9) | 0.16 | -0.08, 0.41 |
| PLT | 187.0(150.0,231.0) | 195.0(166.0,232.0) | -0.12 | -0.31, 0.08 | 191.0(155.0,231.0) | 186.0(165.0,219.0) | 0.09 | -0.15, 0.34 |
| NLR | 2.9(2.0,4.7) | 2.4(1.7,3.9) | 0.25 | 0.05, 0.45 | 2.9(1.9,4.5) | 2.5(1.8,4.0) | 0.24 | -0.01, 0.48 |
| TC | 4.4(3.7,5.0) | 4.3(3.7,4.9) | 0.10 | -0.10, 0.29 | 4.3(3.8,5.1) | 4.2(3.6,4.7) | 0.21 | -0.04, 0.46 |
| TG | 1.3(0.9,1.8) | 1.4(1.0,1.9) | -0.08 | -0.28, 0.12 | 1.2(0.9,1.8) | 1.4(1.0,1.9) | -0.06 | -0.31, 0.19 |
| HDL | 1.1(1.0,1.3) | 1.1(0.9,1.3) | -0.07 | -0.27, 0.12 | 1.1(1.0,1.3) | 1.2(0.9,1.3) | -0.15 | -0.40, 0.10 |
| LDL | 2.6(2.1,3.1) | 2.5(2.0,3.1) | 0.09 | -0.11, 0.28 | 2.6(2.2,3.2) | 2.4(2.0,3.0) | 0.23 | -0.02, 0.47 |
| ALT | 16.4(12.2,22.2) | 16.3(11.7,22.1) | -0.01 | -0.21, 0.18 | 16.4(12.1,23.0) | 15.4(11.7,21.3) | 0.12 | -0.13, 0.37 |
| AST | 18.9(15.6,24.1) | 18.2(15.8,22.5) | 0.06 | -0.13, 0.26 | 18.5(15.0,23.5) | 19.0(16.0,22.0) | 0.12 | -0.12, 0.37 |
| TBIL | 11.7(9.3,15.8) | 11.7(8.4,15.1) | 0.15 | -0.05, 0.35 | 11.3(9.1,14.9) | 12.3(9.0,15.2) | -0.14 | -0.39, 0.11 |
| ALB | 39.5(37.5,41.7) | 40.3(37.8,42.5) | -0.19 | -0.39, 0.01 | 39.5(37.4,41.8) | 40.0(37.8,42.4) | -0.14 | -0.39, 0.11 |
| BUN | 5.7(4.7,7.2) | 5.6(4.4,6.6) | 0.22 | 0.02, 0.41 | 5.5(4.6,7.2) | 5.7(4.4,6.7) | 0.20 | -0.05, 0.45 |
| Cr | 74.0(64.0,90.0) | 72.1(61.0,87.0) | 0.07 | -0.12, 0.27 | 76.0(61.8,89.0) | 76.7(62.1,88.0) | 0.03 | -0.22, 0.28 |
| HCY | 15.8(12.2,23.4) | 13.1(10.5,17.1) | 0.37 | 0.17, 0.57 | 14.9(11.6,21.4) | 14.0(10.5,18.4) | 0.26 | 0.01, 0.51 |
| DD | 0.3(0.2,0.5) | 0.6(0.3,1.1) | -0.43 | -0.63, -0.23 | 0.3(0.2,0.4) | 0.7(0.3,1.2) | -0.43 | -0.68, -0.18 |
| Fib | 3.5(3.0,4.1) | 3.0(2.5,3.6) | 0.63 | 0.43, 0.84 | 3.5(3.0,4.1) | 2.9(2.5,3.5) | 0.69 | 0.44, 0.95 |
| Symptomatic intracranial  hemorrhage at 24 h | 0 (0.0%) | 3 (1.5%) | 0.17 | -0.02, 0.37 | 0 (0.0%) | 1 (0.8%) | 0.13 | -0.12, 0.38 |
| Hemorrhagic  transformation at 24 h | 3 (1.5%) | 6 (3.0%) | 0.10 | -0.10, 0.30 | 2 (1.6%) | 3 (2.4%) | 0.06 | -0.19, 0.31 |
| 1n (%); Median(Q1,Q3) | | | | | | | | |
| 2Standardized Mean Difference; Cohen's D | | | | | | | | |
| Abbreviation: CI = Confidence Interval | | | | | | | | |
